# Risk Assessment and Source Identification of 17 Metals and Metalloids on Soils from the Half-Century Old Tungsten Mining Areas in Lianhuashan, Southern China

**DOI:** 10.3390/ijerph14121475

**Published:** 2017-11-29

**Authors:** Li Guo, Weituo Zhao, Xiaowen Gu, Xinyun Zhao, Juan Chen, Shenggao Cheng

**Affiliations:** 1 School of Environmental Studies, China University of Geosciences, Wuhan 430074, China; guohanli3428@126.com (L.G.); zxyscholar@163.com (X.Z.); cjuan1101@163.com (J.C.); 2 The Center of Environmental Engineering and Assessment, No. 203 Research Institute of Nuclear Industry, Xianyang 712000, China; weituo2006@126.com; 3 School of Earth Sciences, University of Melbourne, Parkville, VIC 3010, Australia; 111111guoli@163.com

**Keywords:** abandoned tungsten mine, metals and metalloids, risk assessment, multivariate geostatistics, chemical fractionation

## Abstract

*Background:* Mining activities always emit metal(loid)s into the surrounding environment, where their accumulation in the soil may pose risks and hazards to humans and ecosystems. *Objective*: This paper aims to determine of the type, source, chemical form, fate and transport, and accurate risk assessment of 17 metal(loid) contaminants including As, Cd, Cu, Ni, Pb, Zn, Cr, Ag, B, Bi, Co, Mo, Sb, Ti, V, W and Sn in the soils collected from an abandoned tungsten mining area, and to guide the implementing of appropriate remediation strategies. *Methods*: Contamination factors (*CFs*) and integrated pollution indexes (*IPIs*) and enrichment factors (*EFs*) were used to assess their ecological risk and the sources were identified by using multivariate statistics analysis, spatial distribution investigation and correlation matrix. *Results*: The *IPI* and *EF* values indicated the soils in the mine site and the closest downstream one were extremely disturbed by metal(loid)s such as As, Bi, W, B, Cu, Pb and Sn, which were emitted from the mining wastes and acid drainages and delivered by the runoff and human activities. Arsenic contamination was detected in nine sites with the highest *CF* values at 24.70 next to the mining site. The Cd contamination scattered in the paddy soils around the resident areas with higher fraction of bioavailable forms, primarily associated with intense application of phosphorus fertilizer. The lithogenic elements V, Ti, Ag, Ni, Sb, Mo exhibit low contamination in all sampling points and their distribution were depended on the soil texture and pedogenesis process. *Conclusions*: The long term historical mining activities have caused severe As contamination and higher enrichment of the other elements of orebody in the local soils. The appropriate remediation treatment approach should be proposed to reduce the bioavailability of Cd in the paddy soils and to immobilize As to reclaim the soils around the mining site. Furthermore, alternative fertilizing way and irrigating water sources are urgencies to reduce the input of Cd and As into the local soils effectively.

## 1. Introduction

Like many other developing countries, such as Congo, Morocco, and Chile, China has been suffering from serious soil pollution caused by metals and metalloids [1,2,3,4]. Metals and metalloids in soils naturally derive from lithogenic sources, but intense industrialization and urbanization in these countries has accelerated the release of these toxic elements into soils from traffic, fuel components, industrial waste dumping, and other non-specific sources [5,6]. Additionally, agricultural activities, mining, and smelting operations represent other important sources of metal(loid)s in local soils [7,8,9,10,11]. Special attention has been paid to the soil contamination produced by ancient mining activities, due to its marked adverse effect on the health of the local population, and potential for remediation and reclamation [12,13,14,15,16].

Determining the contamination source, basic chemistry, and risk assessment of the metals and metalloids in soils is critical for any remediation decision or reclamation strategy proposal [17]. This study focused on an abandoned tungsten mine, which flourished during the years from 1958 to 1999. While a previous study reported severe arsenic contamination in the local water resource, food, and agricultural soils due to the flooding of the acid mining drainages produced by the ancient mining activities [18], there are no published reports describing other common metal pollutants in mining affected soils, such as Pb, Cu, Zn, Cd and Cr, which were also identified as priority control elements in tungsten mine areas [19]. The distribution of the elements, existing in the orebody, is characterized by a high level of heterogeneity in the mine soils [16]. Thus, it is easy to identify whether a metal or metalloid is related with the mining activities or not by using Principal Component Analysis (PCA) to analyze the underlying patterns between contaminants and the orebody elements. Thus, this paper chose 17 elements, which not only include the usual soil pollutants, but also include the elements composing the mineral and other lithospheric elements. The objectives of this study are: (1) to assess the environmental risk on the basis of the vertical and superficial distributions of the 17 metal(loid)s in the soils around the studied abandoned mining area; (2) to establish a general method to identify the metal(loid) sources and pathways in similar ancient mining areas and (3) to determine the potential bioaccumulation ability and natural attenuation mechanism of the common soil pollutants in soils, including Cr, Ni, Cu, Zn, Cd, Pb and As, by sequential extraction tests.

## 2. Materials and Method

### 2.1. Study Area and Soil Sampling Site

**Figure 1:**
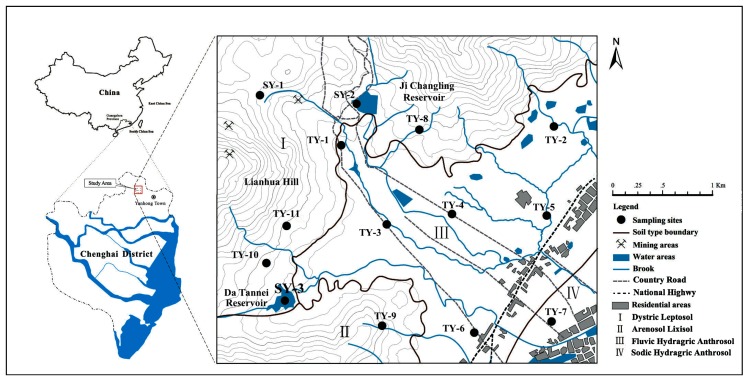
Location map of the study area and sampling sites.

The study area is located in the northeast of Chenghai district, in the center of the Hanjiang delta economic zone of Guangdong Province, China (Figure 1). The area has a characteristic subtropical monsoon climate, with an annual average rainfall of 1444 mm, and temperature of 21 °C, and an east prevailing wind direction. The flourishing ancient mining activities caused extensive damage to the local vegetation and left open pits, mainly distributed in the northern and west sides of the Lianhuashan hills. This study focused on the residential neighborhoods and urban areas, with landforms consisting of alluvial fans along the mountain fronts and the paralic deposited plains. The urban area has been traditionally associated with agricultural activities favoring mainly the production of paddy rice, sugarcane, and guava trees. At places, vegetables like potatoes and tomatoes and fruits like longans and bananas are cultivated. The industry is little developed in the residential neighborhoods, with some small metal processing plants scattered along the national highway.

In order to ensure even sampling, the study area was firstly divided into a grid composed of 12 rectangular cells (1 km × 1 km) and then eleven soil sites (abbreviated by “TY”) were selected within this grid with a cell located in the hilly peak excluded. Sites TY-8–11 were located on the hillside and represented underdeveloped mountain soils. Six sites, including of TY-1, and TY-3–6, were located in the alluvial fans along the mountain fronts, and TY-1 was suspected to be mainly affected by the ancient mining activities. Site TY-7 represents a coastal soil developed from alluvial deposits, with two small metal processing plants on the north side (at approximately 100 m distance). Site TY-2, located in the northwest of the study area and far away from the mining zone, is considered as a relatively less contaminated area. Figure 1 specifies the location and Table 1 lists the geology and soil type details of these sampling sites.

### 2.2. Sample Collection and Analysis

The sampling was carried out in the autumn. At the sites of area one, on the hillside, four topsoil samples were collected at an interval of 10–20 cm below the surface. Seven soil profiles were sampled at the sites located in the urban and residential areas. Three bulk soil samples were collected from each soil layer. Samples were collected at an interval of 10–50 cm (A horizon) for the topsoil layer, 50–100 cm (B horizon) for the subsoil layer, and 100–150 cm (C horizon) for the regolith layer, respectively. These samples were thoroughly mixed into an approximately 5 kg composite sample and then packed in self-locking polyethylene bags. After air-drying at room temperature and passage through a 2 mm nylon sieve, 0.5 g of each soil sample were digested with a mixture of 40% HF-70% HClO_4_-70% HNO_3_ on a hot plate. The digested solution was cooled, filtered and finally diluted to 50 mL. The concentrations of Cu, Ni, Pb, Zn, Cr, Co, Ti, V, As and P were determined by ICP-OES (OPTIMA 8300, PerkinElmer, Waltham, MA, USA), while the contents of trace elements such as Cd, Bi, Mo, Sb, W Ag, Sn and B were detected by ICP-MS (NexION 350, PerkinElmer, Waltham, MA, USA). The 7-step sequential extraction method recommended by the China Geological Survey [21] was used. An initial soil to solution ratio of 1:10 was used and details on each step of the protocol are given in Table S1 of the Supplementary Materials. The choice of extraction of exchangeable, carbonate-bound, Fe and Mn oxide-bound, refractory organic matter-bound, and residual fractions of metal(loid)s in the soils was according to Tessier [22]. The extraction of humic acid-bound fraction was according to Boruvka [23]. The pH was measured in a 1:2.5 soil: water suspension after shaking for 2 h, using a calibrated PB-21 pH meter (Sartorius AG, Goettingen, Germany). The organic matter (OM) concentration was determined by the Walkley-Black method. The cation exchange capacity (CEC) was determined by the sodium acetate saturation method [24].

### 2.3. Contamination Factor (CF) and Integrated Pollution Index (IPI)

The contamination factor (*CF*) was calculated by dividing the metal and metalloid concentration in the soil by their background values, determining the contaminating level from the single one [25]. This study used the background values for the soils of Guangdong Province [26]. The four categories are generally classified by their intensities as suggested by Hakanson [27] and Darwish et al. [28]: low contamination (*CF* < 1), moderate contamination (1 ≤ *CF* < 3), considerable contamination (3 ≤ *CF* < 6), very high contamination (*CF* ≥ 6). The overall pollution status of the metal and metalloid in soils could be assessed by an integrated pollution index (*IPI*) [29], which was defined as:

*IPI* = (*CF*_1_ × *CF*_2_ × *CF*_3_ × ······ × *CF*_n_) ^1/*n*^ (1)

where *n* is the number of samples. Like *CF*, the pollution levels of contaminants were mainly classified into four categories with IPI: no pollution (*IPI* < 1), moderate pollution (1 < *IPI* < 2), heavy pollution (2 < *IPI* < 3), and extremely heavy pollution (3 < *IPI*).

### 2.4. Enrichment Factors (EFs)

Enrichment factors were refined as a means of identifying and quantifying human interference with global element cycles and were widely used in environmental sciences to speculate the origin of elements [30,31]. The enrichment factor (*EF*) of each element can be expressed by the following equation [32,33]:

(2) $$EF=\frac{C_{i}/C_{r}}{B_{i}/B_{r}}$$

where *C_i_* and *C_r_* are the concentrations of the target metal and the reference metal in in soils, while *B_i_* and *B_r_* are their corresponding background concentrations [26]. This study also chose Ti as a reference element due to the fact its concentration is practically exclusively influenced by crustal sources in the sample medium [30,34]. Based on *EF* values, five enrichment categories are recognized: depletion to minimal enrichment (<2), moderate enrichment (2–5), significant enrichment (5–20), very high enrichment (20–40), extremely high enrichment (>40) [31]. In addition, a mobilization or depletion might occur to the metals if *EF* less than 1.0, whereas the elements with *EF* values more than 1.0 might originate from anthropogenic activities [35].

### 2.5. Statistical and Spatial Distribution Analysis

The multivariate statistics analysis and correlation matrix was carried out using SPSS 13.0 (SPSS Inc., Chicago, IL, USA) on the basis of the concentrations of trace elements and phosphorus in surface soils. The ordinary kriging (*OK*) method provided in ArcGIS 9.3 (ESRI, RedLands, CA, USA) was adopted to determine their spatial distribution [36,37]. Using the kriging equations, *OK* weights were deduced by a semivariance function, and equal half of the average squared difference between paired data values [38,39]. Among the models, a spherical semivariance model is more applicable to this study due to its minimum prediction errors when used for spatial interpolations:

(3) $$\gamma (h)=\frac{1}{2N(h)}\sum_{i=1}^{N(h)}[z(x_{i})-z(x_{i}+h){]}^{2},$$

where *γ*(*h*) represents the semivariance value, *N*(*h*) is the total number of sample pairs within the distance interval *h*, *z*(*x_i_* + *h*) and *z*(*x_i_*) are sample values at two points separated by the distance interval *h*, and *x* is the position of soil sample sites.

## 3. Results

### 3.1. Metal Concentrations in Three Different Horizon Soils

Soil properties analyzed in this area are shown in Table 1. The total concentrations of As, Cd, Cu, Ni, Pb, Zn, and the ones of other investigated elements in the top soils are given in Table 2 and Table S2, respectively.

**Figure 2:**
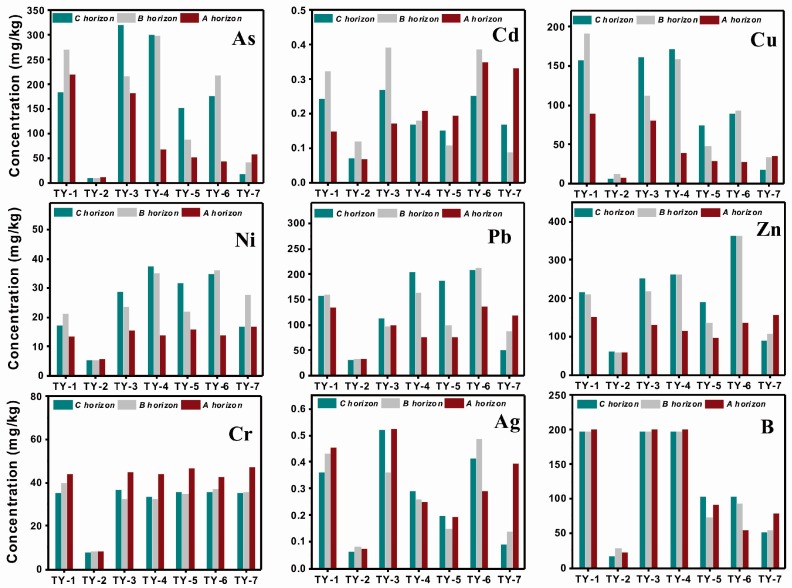

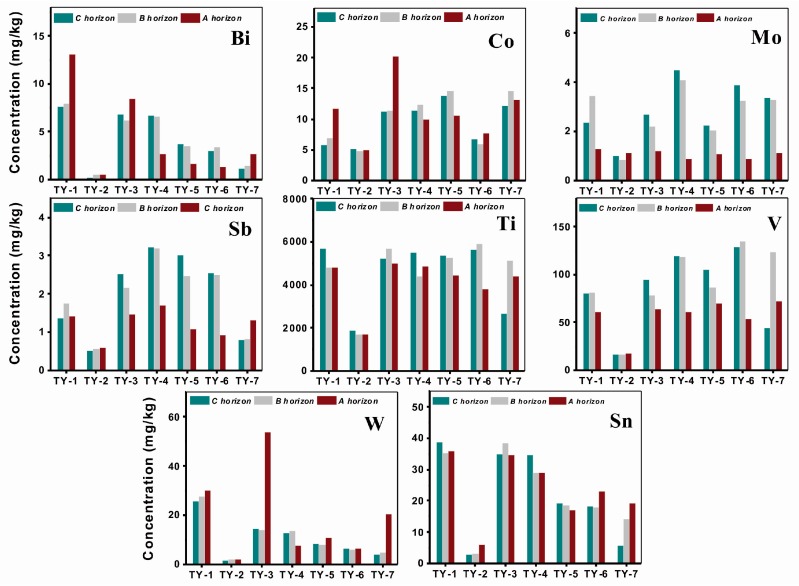
The metal(loid) concentrations in the soils of three different layers.

Figure 2 contrasts the total concentrations of the 17 elements in three different horizon soils from the plain field (including sample sites from TY-1 to TY-7, the total concentrations are shown in Table S4). Among these sites, TY-2, located far away from the mine zone, showed the lowest element concentrations in all the soils at every layer. The elements of W, Co, Bi, Pb, Ag, and Sn exhibited higher contents in the top soils than the two deeper layers of the sites adjoined to the mine zone, except for As, which was prone to enrichment in the deeper soils. However, the fresh inputs of As by the local small metal processing plants elevated the contents in the top soil at site TY-7. In the residential area, the concentrations of Cd decreased with the depth deeper, indicating its human input [41]. Similarly, elevated contents of Zn and Pb detected in surface soil in the residential area may were attributed to human activities. Cu in top soils surrounding the mine zone exhibited higher contents than the levels in other area. Relying on lithogenic sources, elements of B, Cu, Ni, Mo, Sb, Ti and V were mainly enriched in the deeper soils.

### 3.2. Risk Assessment of Metals and Metalloids on Top Soils

#### 3.2.1. CFs and IPIs of Metals and Metalloids in Top Soils

Table 3 summarizes the single contamination factors (*CFs*) and integrated pollution indices (*IPIs*) of the target elements in top soils. The soils collected in the vicinity of the mining area showed very high contamination of As, B, Bi, W and Sn, considerable contamination of Cu, Pb, Zn and Ag, moderate contamination of Cd, Co, Sb and Ti. As and W gave the highest values (24.70 and 41.27, respectively). The above ten elements also exhibited higher contamination in the top soils of the residential area, with considerable contamination of As, Cd, Pb, B, Bi, W and Sn and moderate contamination of Cu, Co, Sb and Ti. Meanwhile, Ag caused various degrees of contamination in the top soils ranging from moderate to considerable contamination. Other elements Ni, V, Cr, Mo exhibit low contamination in all sampling points, indicating their lithogenic sources. The integrated pollution indices (*IPIs*) analysis showed four different classes of all the sites. Sites TY-1 and TY-3 near the mining area had the highest *IPI* (>3). Site TY-11 also had relatively high *IPI.* Soils taken from the residential area (TY-7) and along the downstream near the mine (TY-4) were both heavily polluted. The rest sites (TY-5 and TY-6) in the residential area and the other samples (TY-8 and TY-10) collected from low hilly area all showed moderate pollution of metals and metalloids.

#### 3.2.2. EF Values and Spatial Distribution of Metals and Metalloids in Top Soils

**Figure 3:**
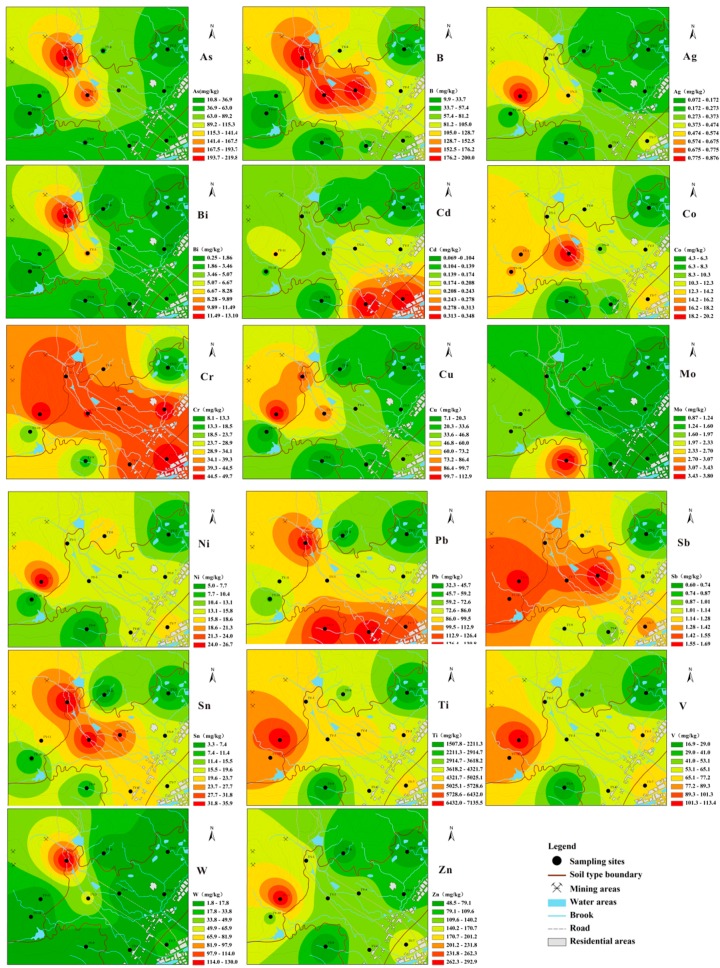
Spatial distribution of metals in the surface soils of the studied area.

Table 1 shows the *EF* values of the metals and metalloids in the top soils. Figure 3 displays their spatial distribution maps, where red colors represent higher concentrations and green colors indicate lower concentrations. According to the metal enrichment factors, the top soils in this area showed significant enrichment of As and W, and moderate enrichment of Bi, B, Sn, Cd and Pb, with a descending order of As > W > Bi > B > Sn > Cd > Pb. Notably, the *EF* values of these metals differed with sample sites, indicating multiple sources. However, As, Bi, W, B, Cu, Pb and Sn showed a very similar spatial pattern (Figure 3), with the higher concentrations found in the mining area and decreasing along the river watershed, representing their release from the mining activities and migration in surface waters. For Pb, another concentration hotspot was also found in the residential area with a uniform concentration pattern along the major roads. The metal Cd was also prone to be concentrated in the residential area.

The metals Ni, Cr, Mo and V exhibited depletion to minimal enrichment over all the studied area, suggesting their lithogenic sources, such as soil texture and pedogenesis in the study area. The metal Mo, produced by a unique source, also showed a unique spatial pattern in the soils with the highest concentration located in the west-north areas, which were characterized geomorphologically as alluvial deposits at the lowest altitudes in the study area.

### 3.3. Source Identification by Multivariate Statistical Analysis

#### 3.3.1. Principle Component Analysis 

Table 4 shows the Principle Component Analysis (PCA) results determined on the basis of the total concentration of the investigated elements in the top soils. The first four principle components (PCs) account for 88.6% of the total variance in the data and were retained for further analysis. Principle component 1 (PC1), explaining 34.2% of the variance, dominated by V, Ti, Zn, Ag, Ni, Sb, Cu and Co, which were identified as lithogenic sources. Principle component 2 (PC2), which has highly positive loading of As, Bi, W, B, Sn and moderate positive loading of Cu and Pb accounts for 27.7% of the variance, which were similarly attributed to mining activities. Copper indicated a mixed source from both lithogenic and anthropogenic input, with moderately correlation to PC1. Principle component 3 (PC3) has highly positive loading of Cd, P, Pb and Cr and accounts for 17.2% of the variance, represented as agricultural sources. Mo is isolated in the fourth component (9.4% of the variance) and highly negatively loaded on PC4.

#### 3.3.2. Cluster Analysis Results

**Figure 4:**
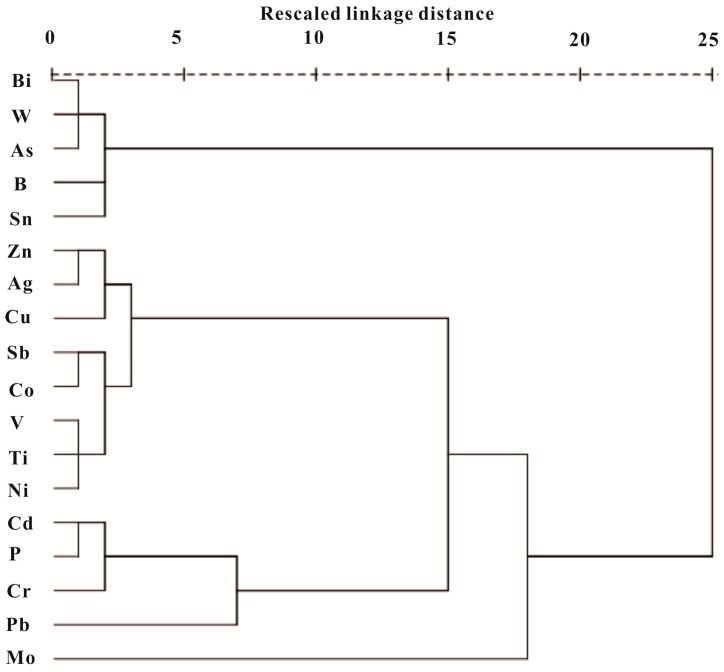
Dendgram of target metals and phosphorus using hierarchical clustering analysis for parameters.

Cluster analysis was also performed using the Ward method to identify similarities in concentrations between the 17 metals and the element P. These elements can be grouped into four main clusters in the dendrogram shown in Figure 4. Cluster 1 included the elements Bi, W, As, B, and Sn that had previously been interpreted as anthropogenic elements ascribed to former uncontrolled mining activities in this region. Cluster 2 contained Zn, Ag, Cu, Sb, Co, V, Ti and Ni, which were also identified as lithogenic sources in the above section. The elements in Cluster 3 including Cd, P, Cr and Pb were mainly related with the intense anthropogenic activities in this area. There was only Mo contained in Cluster 4, implying a different nature source with the ones in Cluster 2, indicated its unique source.

### 3.4. Chemical Fractionations of Toxic Elements in Three Different Layer Soils

**Figure 5:**
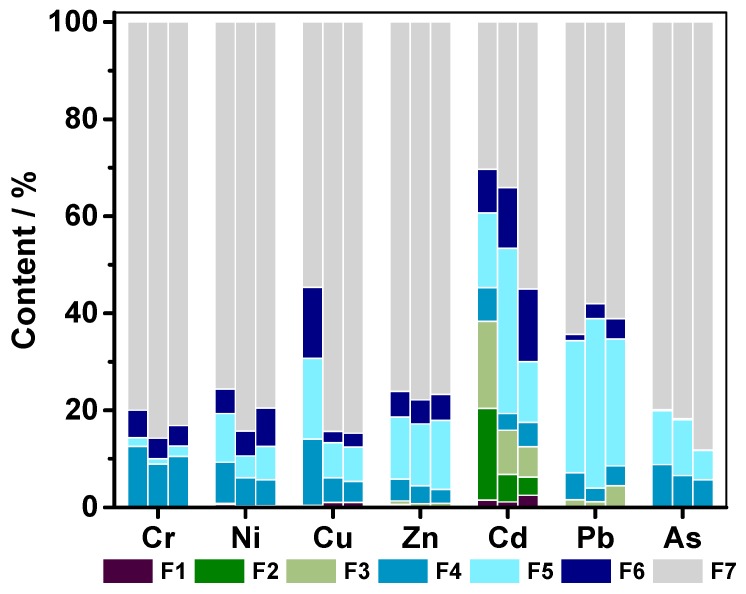
Chemical fractionations of Cr, Ni, Cu, Zn, Cd, Pb and As in surface (see in the left column), middle (see in the middle column) and deep soils (see in the right column) of the study area (F1, F2, F3, F4, F5, F6 and F7 represent water soluble, exchangeable, carbonate-bound, humic acid-bound, Fe and Mn oxide-bound, refractory organic matter-bound, and residual fractions, respectively).

This study investigated the chemical fractionation of the soil pollutant including Cr, Ni, Cu, Zn, Cd, Pb and As, which were found relatively highly enriched in the soils from the studied area. The results are depicted in Figure 5 and each fraction was indicated as a percentage of the sum of all the fractions.

The majority of Cr, Ni, Zn and As was present in the residual fraction (70–88% of the total concentration) in the three different layer of soils. Besides, appreciable amounts of Cu were determined by residual fraction both in the soils of B and C horizon. In top soils, the concentration of Cu in residual fraction accounted for 51.17%, reduced 34.28% compared with the percentage in subsoils. In contrast, the humic acid-bound, Fe and Mn oxide-bound concentration and refractory organic matter-bound of this metal increased from 5.18%, 7.30%, 2.36% in subsoils to 12.78%, 15.58% and 13.7% in top soils, respectively, indicating the strong sorption of Cu to Fe and Mn oxide and organic matter in an oxidizing environment [42]. Pb were mainly presented in the residual fraction (57.89–62.39%) and followed by Fe-Mn oxide-bound fraction (26.04–35.95%) in the soils at every layer. A higher residual fraction concentration of Cd was only found in the deepest soils and decreased with depth. Notably, the concentration of Cd in the first four fractions reach up to 40.62% in top soils, which were considered as mobile and bioavailable ones [43,44]. It can be concluded that this metal has strongly with anthropogenic source in this study area and was prone to transfer from soils to crops and underground water.

## 4. Discussion

### 4.1. Soil Contaminant Risks and Sources

The principal contaminants in the mining area were all in agreement with the mineralogy of the site, and a review reported that the elements of As, Cd, Cr, Cu, Ni, Pb and Zn were prone to enrichment in the soils from the tungsten mining area [19]. Similarly, the contents of the above elements were all elevated in the soils of the studied abandoned tungsten mining area. The situations of As, Cu, Pb, and Cd were more worse in this mining area, due to tact most of their concentrations were above the maximum permitted levels for agricultural soils in China [39]. Dudka and Miller [45] highlighted the arsenic exposure risk to organisms when its concentration exceeds 40 mg/kg. However, the results from this study showed that approximately 73% of the soils were higher than the above permitted level. The lead levels in this area could be considered safe since the highest concentration in sites was below the safe level of 300 ppm [46]. The Cd levels in the residential area were above the permitted levels, with a large amount of mobile and bioavailable forms.

Mining activities have been extensively reported as a major source for metals and arsenic in local soils [43,47,48,49]. In the soils of this studied area, the hotspots of the elements As, Bi, W, B, Sn, Pb and Cu were found to correlate well with the spread of mining and processing activities. Furthermore, the cluster analysis results only confirmed the common source of first five elements but not for the last two ones, although the moderate positive loading of Pb and Cu in PC2 and the moderate correlations with W could also show their sources from the mining activities. Another group of anthropogenic elements, including Cd, P, Pb and Cr, should be attributed to intense anthropogenic activities, with concentration hotspots in the residential area. Table S3 showed the higher contents of Pb, Cd, Cr, and P in one of the phosphorus fertilizer collected from the local market. Thus, the above elements were primarily associated with intense application of phosphorus fertilizer, proved in previous works [28,41,50,51,52,53,54]. The abusing of fertilizers and manure elevated the content of Cr in the surface soils of residential areas [55]. The enrichment of Pb in this region was also highly related with traffic emissions, with higher enrichment in the top soils along the national highway, and a marked deposit in the hilly area down the wind.

The natural source elements were also grouped into two categories by the integrated methods. The first one including the elements of V, Ti, Zn, Ag, Ni, Sb, Cu and Co, were highly influenced by soil texture and pedogenesis in the study area. Granite weathering profiles characterized by a high content of oxide Al and Fe clay minerals are widespread in Southern China [56]. Due to the high affinity and adsorption capacity of the above metals, clay minerals in the soils are mainly responsible for their distribution [57]. For instance, Ti always exists as ultrafine particles of TiO_2_ that are associated with the clay fraction or co-precipitate with iron oxy (hydro) oxides in soils [28,58]. As well-known siderophile elements, Co, Ni, Ti, V, Ag and Zn were prone to retention or enrichment in soils by adsorbing or incorporating in stable Fe oxides [59,60,61,62,63] and re more likely to be found in granite latosolic red soils which hold more micronutrients and water than sandy soils [41]. In addition, samples collected in TY-11 where the soil was lying directly on the in-situ weathered or decomposed rock exhibited the highest enrichment for V, Ti, Zn, Ag, Ni, Cu and Co and the second higher content for Sb compared with other sites, again confirmed their lithogenic origin. The other category only contained the metal of Mo with a content hotspot existing in the alluvial areas, indicating it derived from alluvial deposits.

### 4.2. Fate and Transport of the Metals and Metalloids

The fate and transport of metals and metalloids in the soils in this area were mainly dependent on their chemical forms and human activities. During a series of mining processes, the metals and metalloids co-existing with the target minerals, including As, Bi, W, B, Sn, Pb and Cu, always transfer into the atmospheric particulate matters, mining wastes and acid mine drainages. Naturally, these emitted atmospheric particulate matters were hardly delivered for a long distance and easily settled down near to the mining centers [64]. However, during the rainy season, the metals and metalloids in the wastes and settled particles along with the acid mine drainages could easily enter into the main drainage ditches. In this area, the Ji Changling Reservoir is still polluted by arsenic and showed higher Zn, Cu, Pb concentrations in the surface water than the other reservoir (seen in Figure S1 of the Supplementary Materials). Due to the long term irrigation, these metals and metalloids have caused higher accumulations in the downstream soils. Compared with others, arsenic is predominantly in anionic forms in the aquatic system, thus it produced a wider and higher pollution degree in the downstream region [65]. The abuse of application of phosphorus fertilizer, manure and pesticides has introduced Cd, Cr and Pb into the paddy soils. Furthermore, the crude recycling processes should be responsible for the distribution of Pb and As in the soils from residential area.

Once entered into the soils, these metals and metalloids are always fixed by iron (Fe) oxyhydroxides, carbonates and soil organic matters, and then transferred into different chemical forms with varying bioavailability, mobility and toxicity [17]. According to the sequential extraction results, the binding with humic acids, Fe and Mn oxides and refractory organic matters are the main processes for Cr, Ni, Cu, Zn retained in the studied soils. Carbonates also play an important role to fix Cd and Pb, except for the above three main bonding processes. Among them, the concentrations of the water soluble and exchangeable species of Cd were detected in all three layers of soils, which are easily transported and absorbed by organisms and thereby pose a high hazard potential to the environment [66]. Arsenic could be easily adsorbed by humic acids and Fe and Mn oxides due to its acid ion forms. However, the fixation process became weaker with the deeper of the soil layers, attributed to the weaker affiliation of reduced As species on these active surfaces which is the predominant forms in the reducing conditions.

### 4.3. Remediation and Reclaim Implementations

The contaminants of Cd, As and Pb should be taken into consideration as the target elements for the regulatory authorities to implement remediation strategies, based on the assessment of the type and level of contamination in the studied soils. Source control and containment remedies are the two main technologies to remediate or to reclaim the contaminated soils [67]. The intense anthropogenic activities are still active sources for Pb and Cd into the soils, thus, the fertilizer should be properly used. Furthermore, the regulatory authorities should enhance the monitoring for the local metal recycling factories to eliminate fresh emissions of Pb and As. Furthermore, the appropriate remediation treatment approach should be proposed to reduce the bioavailability of Cd. Effective amendments of lime or fly ashes from thermal power plants for the immobilization of Cd could be suitable for the contaminated paddy soils [68] and hyperaccumulating plants, such as *Thlaspi caerulescens* and *Arabidopsis halleri* with higher accumulation for Cd, could be used to reclaim the wasteland [69]. Soil aging (natural attenuation) effectively transferred most bioavailable fractions of As into the residual ones, due to its historical and discontinuous origin [70]. Thus, a remediation approach is not an urgent need in the paddy soils, but should be implemented at the mining site due to the severe contamination caused by arsenic. The irrigation with the local surface water should be forbidden until the quality of the water body reaches the permitted level.

## 5. Conclusions

The distributions, sources, and pathways of 17 metal(loid)s in soils surrounding an abandoned tungsten mine were investigated. The Integrated Pollution Index (*IPI*) analysis showed four different classes of the sample sites in the study area: no pollution sites (TY-2 and TY-9), moderate pollution sites (TY-5, TY-6, TY-8 and TY-10), heavy pollution sites (TY-4, TY-7, and TY-11), and the extremely heavy pollution sites TY-1 and TY-3, with higher enrichment of W, significant enrichment of As, Bi, B, moderate enrichment of Cd, Cu, Pb, Zn, Ag. These elements were mainly originated from mineral paragenesis and ancient mining activities. The long term irrigation using polluted surface waters (Ji Changling Reservoir), have caused higher accumulation of these elements in the downstream agricultural soils. Compared with others, arsenic is more mobile, thus, it produced a wider and higher pollution in the downstream soils. The intense anthropogenic activities were responsible for the enrichment of Cd, Pb and Cr in the residential soils. Among them, Cd displayed higher mobility and was easily released into local soil and underground water which deserve more attention by the government when implementing remediation programs. The local small metal processing plants still emitted As into the surroundings and should be supervised. Heavy traffic and prevailing wind determined the deposition of Pb in top soils at TY-6, TY-7, and TY-9. Effective treatment should be used to immobilize As before reclaiming the soils around the mining site. Furthermore, alternative fertilizing methods and irrigation water sources are urgent needs to effectively reduce the input of Cd and As into the local soils.

## Figures and Tables

**Table 1:** The site geology and physicochemical properties of top soils in the studied area.

Site	Geology	Soil Classification ^a^	Land Use	pH	CEC (meq/100 g)	OM (%)
TY-1	Alluvial clay and gravel ($Q_{4}^{3\mathrm{apl}}$)	Fluvic Hydragric Anthrosol	Wild grass	3.9	16.32	1.23
TY-2	Alluvial clay and gravel ($Q_{4}^{3\mathrm{apl}}$)	Fluvic Hydragric Anthrosol	Paddy	4.2	17.10	3.67
TY-3	Alluvial clay and gravel ($Q_{4}^{3\mathrm{apl}}$)	Fluvic Hydragric Anthrosol	Vegetable field	4.5	16.52	3.86
TY-4	Alluvial clay and gravel ($Q_{4}^{3\mathrm{apl}}$)	Fluvic Hydragric Anthrosol	Vegetable field	4.9	17.26	3.72
TY-5	Alluvial clay and gravel ($Q_{4}^{3\mathrm{apl}}$)	Fluvic Hydragric Anthrosol	Paddy	6.2	17.32	3.62
TY-6	Alluvial clay and gravel ($Q_{4}^{3\mathrm{apl}}$)	Fluvic Hydragric Anthrosol	Paddy	5.7	16.28	3.58
TY-7	Paralic deposits ($Q_{4}^{3mc}$)	Sodic Hydragric Anthrosol	Paddy	6.8	18.96	3.46
TY-8	Late Jurassic monzonitic granites (J_3ηγ_)	Arenosol Lixisol	Forest field	5.8	17.61	2.96
TY-9	Triassic sandy shales and sandy conglomerate (K_1ηo_)	Dystric Leptosol	Forest field	4.7	16.48	2.67
TY-10	Early Cretaceous Epoch quartzdiorites (T_3G_)	Arenosol Lixisol	Forest field	5.1	16.52	2.98
TY-11	Early Cretaceous Epoch quartzdiorites (T_3G_)	Arenosol Lixisol	Forest field	4.9	16.21	3.00

^a^ According to the World Reference Base for Soils [20]; CEC: Cation Exchange Capacity; OM: Organic Matter.

**Table 2:** Concentrations and enrichment factors (*EFs*) of metal(loid)s in top soils from the study area.

Sample	As		Cd		Cu		Ni		Pb		Zn	
	Concentration (mg/kg)	*EF*	Concentration (mg/kg)	*EF*	Concentration (mg/kg)	*EF*	Concentration (mg/kg)	*EF*	Concentration (mg/kg)	*EF*	Concentration (mg/kg)	*EF*
TY-1	219.8 ± 0.7	19.64	0.149 ± 0.003	2.12	88.7 ± 1.2	4.15	13.6 ± 0.3	0.75	133.5 ± 1.2	2.95	150.7 ± 1.4	2.53
TY-2	10.8 ± 0.2	2.06	0.069 ± 0.002	2.09	7.4 ± 0.1	0.74	5.6 ± 0.4	0.66	32.3 ± 0.7	1.52	59.6 ± 0.8	2.14
TY-3	181.8 ± 0.4	11.90	0.171 ± 0.002	1.78	80.9 ± 0.9	2.77	15.5 ± 0.2	0.63	99.0 ± 0.6	1.60	131.2 ± 0.6	1.62
TY-4	68.5 ± 0.8	4.60	0.209 ± 0.009	2.23	39.3 ± 0.5	1.38	13.8 ± 0.3	0.57	75.8 ± 1.6	1.26	116.4 ± 0.8	1.47
TY-5	52.4 ± 0.7	3.86	0.193 ± 0.002	2.26	28.8 ± 0.4	1.11	15.8 ± 0.1	0.72	76.2 ± 1.3	1.39	97.1 ± 0.9	1.35
TY-6	43.8 ± 0.2	3.78	0.348 ± 0.001	4.77	27.7 ± 0.3	1.25	13.7 ± 0.5	0.73	136.8 ± 0.7	2.92	137.3 ± 0.8	2.23
TY-7	57.8 ± 0.8	4.28	0.333 ± 0.004	3.92	34.6 ± 0.6	1.34	16.8 ± 0.2	0.77	118.1 ± 0.9	2.16	156.4 ± 0.9	2.18
TY-8	61.6 ± 0.3	5.77	0.099 ± 0.001	1.47	11.4 ± 0.3	0.56	17.4 ± 0.4	1.01	42.4 ± 0.3	0.98	61.8 ± 0.6	1.09
TY-9	35.9 ± 0.2	7.76	0.072 ± 0.001	2.47	9.2 ± 0.6	1.04	5.0 ± 0.1	0.67	139.8 ± 1.1	7.47	48.5 ± 0.4	1.97
TY-10	18.1 ± 0.4	1.07	0.136 ± 0.002	1.28	7.1 ± 0.3	0.22	6.3 ± 0.1	0.23	58.2 ± 0.6	0.85	130.3 ± 0.8	1.45
TY-11	49.2 ± 0.3	2.25	0.213 ± 0.003	1.55	112.9 ± 1.2	2.70	26.7 ± 0.2	0.75	85.3 ± 0.8	0.96	292.9 ± 1.5	2.52
Max	219.8	19.64	0.348	4.77	112.9	4.15	26.7	1.01	139.8	7.47	292.9	2.53
Min	10.8	1.07	0.069	1.28	7.1	0.22	5.0	0.23	32.3	0.85	48.5	1.09
Mean	72.7	6.09	0.181	2.36	40.7	1.57	13.6	0.68	90.7	2.19	125.6	1.87
SD	66.23	5.41	0.093	1.07	36.8	1.17	6.3	0.19	38.0	1.90	67.1	0.50
BV ^a^	8.9		0.056		17.0		14.4		36		47.3	
MAC ^b^	25		0.30		100		50		300		250	

BV: background value; MAC: maximum allowable concentration. ^a^ background values of elements in the soils of Guangdong Province (CNEMC, China National Environmental Monitoring Center, 1990 [27]); ^b^ The maximum allowable concentrations of contaminants in Chinese soils (EPAC, Environmental protection Administration of China, 2008 [40]).

**Table 3:** Metal contamination factors (*CFs*) and pollution load indexes (*IPIs*) for metals in top soils from the study area.

Sample	Contamination Factors (*CFs*)																	*IPIs*
	As	Cd	Cu	Ni	Pb	Zn	Cr	Ag	B	Bi	Co	Mo	Sb	Ti	V	W	Sn	
TY-1	24.70	2.66	5.21	0.94	3.71	3.19	0.86	4.20	9.17	24.26	1.67	0.16	2.61	1.65	0.92	41.27	6.19	3.36
TY-2	1.21	1.23	0.43	0.39	0.89	1.26	0.16	0.67	1.01	0.98	0.71	0.15	1.11	0.59	0.26	0.57	1.00	0.62
TY-3	20.43	3.05	4.76	1.07	2.75	2.77	0.89	4.84	9.17	15.68	2.88	0.16	2.70	1.72	0.98	17.07	5.96	3.16
TY-4	7.69	3.73	2.31	0.96	2.11	2.46	0.87	2.31	9.17	4.93	1.43	0.11	3.13	1.67	0.92	2.44	4.98	2.10
TY-5	5.89	3.44	1.69	1.09	2.12	2.05	0.92	1.79	4.20	3.11	1.50	0.14	2.0	1.52	1.07	3.39	2.91	1.81
TY-6	4.92	6.21	1.63	0.95	3.80	2.90	0.84	2.69	2.47	2.46	1.10	0.11	1.69	1.30	0.81	2.0	3.98	1.74
TY-7	6.49	5.95	2.03	1.17	3.28	3.31	0.93	3.65	3.63	4.93	1.89	0.14	2.43	1.52	1.11	6.48	3.31	2.30
TY-8	6.93	1.77	0.67	1.21	1.18	1.31	0.74	1.39	4.10	0.46	1.64	0.19	2.24	1.20	0.72	0.77	0.57	1.13
TY-9	4.03	1.29	0.54	0.35	3.88	1.02	0.33	0.88	0.53	2.07	0.61	0.49	1.93	0.52	0.29	0.83	1.67	0.90
TY-10	2.03	2.43	0.42	0.44	1.62	2.75	0.37	1.50	0.45	0.87	2.04	0.25	2.85	1.90	1.30	0.65	1.19	1.05
TY-11	5.53	3.80	6.64	1.85	2.37	6.19	0.98	8.11	2.49	4.83	2.07	0.25	2.98	2.46	1.74	2.55	3.57	2.70

**Table 4:** Principal component loading (varimax normalization) for metals of top soils in the study area.

Parameter	PC1	PC2	PC3	PC4
V	**0.937**	−0.052	0.188	0.098
Ti	**0.937**	0.124	0.129	0.172
Zn	**0.864**	0.044	0.384	−0.122
Ag	**0.824**	0.282	0.358	−0.136
Sb	**0.783**	0.286	−0.104	0.037
Ni	**0.753**	0.051	0.377	0.219
Co	**0.742**	0.331	−0.049	0.265
Cu	**0.709**	0.556	0.246	−0.114
As	0.165	**0.969**	0.039	0.061
Bi	0.175	**0.966**	0.070	−0.044
W	0.077	**0.934**	0.039	−0.057
B	0.184	**0.819**	0.037	0.435
Sn	0.280	**0.789**	0.431	0.137
Cd	0.236	−0.077	**0.885**	0.294
P	0.438	0.169	**0.841**	0.120
Pb	−0.104	0.433	**0.694**	−0.499
Cr	0.553	0.332	**0.560**	0.349
Mo	−0.091	−0.154	−0.254	**−0.900**
Eigenvalues	6.164	4.998	3.089	1.704
% of variance	34.244	27.767	17.163	9.464
Cumulative %	34.244	62.012	79.175	88.639

The bold indicate: the significance of a parameter in a specific principle component. Significant at *p* < 0.05.

## Supplementary Materials

The following are available online at www.mdpi.com/1660-4601/14/12/1475/s1, Figure S1: The contents of metal(loid)s and in three surface waters and their maximum allowable concentrations (MAC) used for agricultural irrigation, Table S1. Sequential extraction methods for metal(loid)s in different depth soils from the study area, Table S2. Concentrations and enrichment factors (*EFs*) of metal(loid)s in top soils from the study area, Table S3. Major constituents (mg/kg) of the common chemical fertilizers and pesticides from the study area, Table S4. Total concentrations of trace elements in three different soil layers. Table S1 details the steps and conditions of the sequential extraction used to analyze the fractions of various metals in soils. The concentrations of the investigated elements including Cr, Ag, B, Bi, Co, Mo, Sb, Ti, V, W, and Sn in top soils are shown in Table S2. Table S3 shows the contents of Pb, Cd, Cr, and P in the phosphorus fertilizer and pesticides collected from the local market. Figure S1 shows the contents of metal(loid)s in three surface waters and their maximum allowable concentrations (MAC) in water used for agricultural irrigation.
