# Supplementary material for: Risk Assessment and Source Identification of 17 Metals and Metalloids on Soils from the Half-Century Old Tungsten Mining Areas in Lianhuashan, Southern China
Source: Int J Environ Res Public Health. 2017 Nov 29;14(12):1475. doi: 10.3390/ijerph14121475 (PMC5750894; doi:10.3390/ijerph14121475)
Supplement: Supplementary file 1 [file ijerph-14-01475-s001.pdf]

Supplementary Materials

Figure 1 showed the contents of metal(loid)s in three surface waters and their maximum allowable concentrations (MAC) used for agricultural irrigating. Sample SY-1 (surface water sampling site were abbreviated by “SY”) was collected from the reservoir which is located in the upstream of the mining area. Site SY-2 is located in the Ji Changling reservoir which is close to the downstream of the mining area. Site SY-3 is also located in the downstream of the mining area but is farther from the mine zone, which is close to the residential areas. The surface water of Jichangling reservoir has been polluted by arsenic, and also showed higher Zn, Cu, Pb concentrations than the ones of the other reservoir.

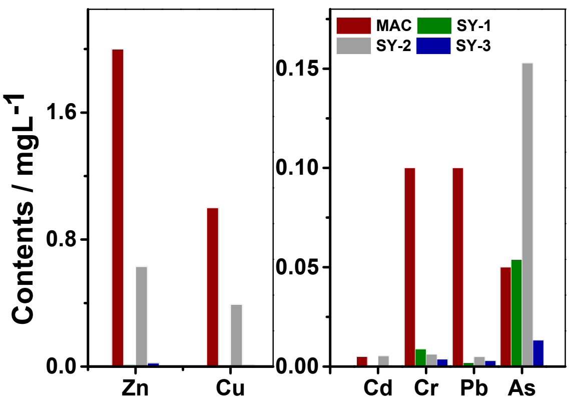

Figure S1. The contents of metal(loid)s and in three surface waters and their maximum allowable concentrations (MAC) used for agricultural irrigating.

Table S1. Sequential extraction methods for metal(loid)s in different depth soils from the study area

Step	Fractions	Extract composition	pH	Notes
**F_1**	Water soluble	25 mL ultrapure water	pH=8.0 ± 0.2	30 min ultrasound (40 KHz, 25 ±5°C), rinse with ultrapure H_2O.
**F_2**	Exchangeable	25 mL 1.0 mol/L $MgCl_2 \cdot 6H_2O$	pH=7.0 ± 0.2	30 min ultrasound (40 KHz, 25 ± 5°C), rinse with ultrapure H_2O.
**F_3**	Carbonate-bound	25 mL 1.0 mol/L $CH_3COONa \cdot 3H_2O$	pH=5.0 ± 0.2	60 min ultrasound (40 KHz, 25 ± 5°C), Rinse with ultrapure H_2O.
**F_4**	Humic acid-bound	50 mL 0.1 mol/L $Na_4PO_7 \cdot 10H_2O$	pH=10.0 ± 0.2	40 min ultrasound (40 KHz, 25 ±5°C), rinse with ultrapure H_2O.
**F_5**	Fe and Mn oxide-bound	50 mL 0.25 mol/L $HONH_3Cl$-HCl		1 h ultrasound (40 KHz, 25 ± 5°C), rinse with ultrapure H_2O.
**F_6**	Refractory organic matter-bound	3 mL HNO_3+5 mL 30% H_2O_2	pH=2.0±0.2	1.5 h bath (83 °C, stirred every 10 min), another 1.0 h bath with 3 mL 30% H_2O_2 (83 °C, stirred every 10 min), rinse with ultrapure H_2O.
**F_7**	Residual	5 mL mixture of 37%HCl -70%$HClO_4$ -70%HNO_3(1:1:1)/5 mL 40%HF		Digested at 105 °C for 3h.

Table S2. Concentrations and enrichment factors (*EFs*) of metal(loid)s in top soils from the study area

Sample	Cr		Ag		B		Bi		Co		Mo	
	Concentration (mg/kg)	*EF*	Concentration (mg/kg)	*EF*	Concentration (mg/kg)	*EF*	Concentration (mg/kg)	*EF*	Concentration (mg/kg)	*EF*	Concentration (mg/kg)	*EF*
TY-1	43.7 ± 0.8	0.69	0.454 ± 0.003	3.34	200.0 ± 1.2	7.30	13.10 ± 0.07	19.30	11.7 ± 0.3	1.33	1.29 ± 0.03	0.13
TY-2	8.1 ± 0.2	0.27	0.072 ± 0.004	1.13	22.0 ± 0.4	1.71	0.53 ± 0.01	1.67	5.0 ± 0.2	1.21	1.13 ± 0.02	0.25
TY-3	44.9 ± 0.3	0.52	0.523 ± 0.005	2.82	200.0 ± 0.9	5.34	8.47 ± 0.08	9.14	20.2 ± 0.5	1.68	1.21 ± 0.04	0.09
TY-4	44.0 ± 0.2	0.52	0.250 ± 0.004	1.38	200.0 ± 0.8	5.49	2.66 ± 0.05	2.95	10.0 ± 0.2	0.85	0.89 ± 0.03	0.07
TY-5	46.6 ± 0.8	0.61	0.194 ± 0.003	1.18	91.6 ± 0.6	2.76	1.68 ± 0.04	2.04	10.5 ± 0.3	0.98	1.07 ± 0.03	0.09
TY-6	42.4 ± 0.3	0.64	0.291 ± 0.002	2.07	53.8 ± 0.3	1.89	1.33 ± 0.04	1.89	7.7 ± 0.2	0.84	0.87 ± 0.02	0.09
TY-7	47.3 ± 0.4	0.62	0.394 ± 0.002	2.40	79.1 ± 0.2	2.39	2.66 ± 0.04	3.24	13.2 ± 0.6	1.24	1.11 ± 0.02	0.09
TY-8	37.4 ± 0.3	0.62	0.150 ± 0.002	1.16	89.5 ± 0.6	3.42	0.25 ± 0.02	0.39	11.5 ± 0.1	1.37	1.48 ± 0.04	0.16
TY-9	16.9 ± 0.1	0.64	0.095 ± 0.005	1.69	11.5 ± 0.1	1.01	1.12 ± 0.01	3.99	4.3 ± 0.3	1.18	3.80 ± 0.08	0.95
TY-10	18.6 ± 0.1	0.19	0.162 ± 0.002	0.79	9.9 ± 0.3	0.24	0.47 ± 0.01	0.46	14.3 ± 0.3	1.07	1.89 ± 0.04	0.13
TY-11	49.7 ± 0.2	0.40	0.876 ± 0.006	3.30	54.3 ± 0.4	1.01	2.61 ± 0.06	1.96	14.5 ± 0.4	0.84	1.91 ± 0.02	0.10
Max	49.7	0.69	0.876	3.34	200	7.30	13.10	19.30	20.2	1.68	3.8	0.95
Min	8.1	0.19	0.072	0.79	9.9	0.24	0.25	0.39	4.3	0.84	0.87	0.07
Mean	36.3	0.52	0.314	1.93	91.9	2.96	3.17	4.27	11.17	1.15	1.51	0.20
SD	14.5	0.16	0.237	0.91	74.9	2.22	4.00	5.52	4.54	0.26	0.83	0.25
BV [a]	50.5		0.108		21.8		0.54		7.0		7.7	
MAC [b]	300		---		---		---		---		---	

BV: background value; MAC: maximum allowable concentration.

[a] background values of elements in the soils of Guangdong Province (CNEMC, China National Environmental Monitoring Ceter, 1990 [27]),

[b] The maximum allowable concentrations of contaminants in Chinese soils (EPAC, Environmental protection Administration of China, 2008 [41]).

Table S2. (*Continued*) Concentrations and enrichment factors (*EFs*) of metal(loid)s and phosphorus in top soils from the study area.

Sample	Sb		Ti		V		W		Sn		P
	Concentration (mg/kg)	*EF*	Concentration (mg/kg)	*EF*	Concentration (mg/kg)	*EF*	Concentration (mg/kg)	*EF*	Concentration (mg/kg)	*EF*	Concentration (mg/kg)
TY-1	1.41 ± 0.04	2.08	4790.1 ± 7.6	1.31	60.5 ± 0.9	0.74	130.0 ± 0.5	32.83	35.9 ± 0.6	4.92	942.1 ± 1.0
TY-2	0.60 ± 0.06	1.89	1708.2 ± 5.3	1.00	16.9 ± 0.3	0.44	1.8 ± 0.05	0.96	5.8 ± 0.3	1.70	199.8 ± 1.5
TY-3	1.46 ± 0.03	1.57	4979.0 ± 6.2	1.00	64.0 ± 0.8	0.57	53.8 ± 0.6	9.95	34.6 ± 0.5	3.47	1430.1 ± 1.5
TY-4	1.69 ± 0.05	1.87	4850.2 ± 3.2	1.00	60.2 ± 0.4	0.55	7.7 ± 0.4	1.46	28.9 ± 0.4	2.98	772.3 ± 1.6
TY-5	1.08 ± 0.01	1.31	4419.0 ± 4.0	1.00	69.8 ± 0.2	0.70	10.7 ± 0.3	2.23	16.9 ± 0.3	1.91	909.4 ± 1.4
TY-6	0.92 ± 0.06	1.30	3776.8 ± 3.2	1.00	53.2 ± 0.5	0.63	6.3 ± 0.4	1.54	23.1 ± 0.2	3.06	1793.9 ± 1.5
TY-7	1.31 ± 0.03	1.60	4402.6 ± 5.6	1.00	72.5 ± 0.6	0.73	20.4 ± 0.5	4.27	19.2 ± 0.8	2.18	1451.2 ± 1.3
TY-8	1.21 ± 0.03	1.87	3479.5 ± 5.6	1.00	47.3 ± 0.6	0.60	2.4 ± 0.2	0.64	3.3 ± 0.2	0.47	464.8 ± 1.3
TY-9	1.04 ± 0.02	3.70	1507.8 ± 6.7	1.00	19.2 ± 0.3	0.57	2.6 ± 0.2	1.59	9.7 ± 0.2	3.22	374.3 ± 0.9
TY-10	1.54 ± 0.04	1.50	5518.2 ± 3.6	1.00	84.7 ± 0.6	0.68	2.0 ± 0.1	0.34	6.9 ± 0.2	0.63	479.6 ± 1.2
TY-11	1.61 ± 0.03	1.21	7135.5 ± 8.9	1.00	113.4 ± 0.5	0.71	8.0 ± 0.4	1.04	20.7 ± 0.4	1.45	1496.9 ± 1.0
Max	1.69	3.70	7135.5	1.31	113.4	0.74	130	32.83	35.9	4.92	1793.9
Min	0.60	1.21	1507.8	1.00	16.9	0.44	1.79	0.34	3.3	0.47	199.8
Mean	1.26	1.81	4233.3	1.03	60.1	0.63	22.3	5.17	18.6	2.36	937.7
SD	0.33	0.69	1611.3	0.09	27.3	0.09	38.7	9.57	11.4	1.32	511.3
BV [a]	0.54		2900		65.3		3.15		5.8		---
MAC [b]	---		---		---		---		---		---

BV: background value; MAC: maximum allowable concentration.

[a] background values of elements in the soils of Guangdong Province (CNEMC, China National Environmental Monitoring Ceter, 1990 [27]),

[b] The maximum allowable concentrations of contaminants in Chinese soils (EPAC, Environmental protection Administration of China, 2008 [41]).

Table S3. Major constituents (mg/kg) of the common chemical fertilizers and pesticides from the study area.

**Type**	**Description**	**Unit**	**As**	**Cu**	**Pb**	**Zn**	**Cd**	**Ni**	**Cr**	**Ca**	**Mn**	**K**	**Mg**	**N**	**P**
**Chemical fertilizers**	Kalium chloratum	mg/kg	<0.5	1.5	<0.1	2.2	0.6	8.0	1.3	0.8	2.1	2.7×10^5	505	3.2	2.7
	Carbamide	mg/kg	<0.5	<1.0	<0.1	<0.5	0.04	2.4	<1.0	0.4	1.2	302.5	5.3	4.3×10^5	1.4
	Superphosphate	mg/kg	2.0	85.8	3.3	58.3	2.6	25.8	10.5	7.5	315	2.2×10^3	1.5×10^4	19.5	6.0×10^4
**Pesticides**	Buprofezin	mg/kg	1.0	13.2	<0.1	40.5	1.0	32.0	1.5	8.6	95	1.5×10^3	20.9	2.4×10^3	401.1
	Yeshuangqing	mg/kg	1.0	2.6	<0.1	10.5	1.2	2.1	2.6	0.2	103.1	3.1×10^3	173.3	1.3×10^4	762.7
	Bishuangling	mg/kg	0.6	8.4	<0.1	14.5	1.6	14.5	<0.5	2.3×10^5	17.7	271.6	311.0	2.9×10^4	1.6×10^2
	Lorsban	mg/kg	<0.5	2.1	<0.1	1.0	0.1	4.2	<0.5	400	0.6	0.7	12.4	8.7×10^3	10.3×10^3
	Suihua 203 emulsion	mg/L	<0.5	<1.0	<0.1	6.0	0.2	3.1	2.2	0.04	0.5	ND	8.9	213.7	159.5
	Acephate	mg/L	<0.5	<1.0	<0.1	<0.5	0.4	3.6	1.0	0.006	0.5	ND	0.7	2.8×10^3	4.8×10^3
	Hipro sulfur phosphorus	mg/L	<0.5	<1.0	<0.1	3.1	0.04	0.1	1.0	0.08	0.1	9.4	9.4	7.5×10^3	27.6
	Emamectin benzoate	mg/L	<0.5	<1.0	<0.1	1.6	<0.01	<0.05	1.0	0.09	0.2	60.5	48.4	3.2×10^3	12.4

ND-not detected.

Table S4. Total concentrations of trace elements in three different soil layers.

Element	Soil layer	TY-1	TY-2	TY-3	TY-4	TY-5	TY-6	TY-7
**As**	**A horizon**	219.8	10.8	181.8	68.5	52.4	43.8	57.8
	**B horizon**	271.2	9.7	216.2	298	87.5	218.6	40.9
	**C horizon**	183.6	10	320.3	300.2	151.2	176	17.8
**Cd**	**A horizon**	0.149	0.069	0.171	0.209	0.193	0.348	0.333
	**B horizon**	0.324	0.12	0.392	0.18	0.109	0.386	0.087
	**C horizon**	0.242	0.07	0.27	0.17	0.151	0.252	0.168
**Cu**	**A horizon**	88.7	7.4	80.9	39.3	28.8	27.7	34.6
	**B horizon**	191.1	12.3	112.5	158.6	47.6	93.7	33.5
	**C horizon**	157.3	5.6	161.2	171.3	74.5	89.5	16.8
**Ni**	**A horizon**	13.6	5.6	15.5	13.8	15.8	13.7	16.8
	**B horizon**	21.2	5.2	23.7	35.2	21.8	36	27.8
	**C horizon**	17.1	5.3	28.6	37.6	31.6	34.8	16.9
**Pb**	**A horizon**	133.5	32.3	99	75.8	76.2	136.8	118.1
	**B horizon**	159.8	33.1	97.8	163	99.8	212.3	87.8
	**C horizon**	157.6	30.5	112	203.5	186.7	207.4	49.7
**Zn**	**A horizon**	150.7	59.6	131.2	116.4	97.1	137.3	156.4
	**B horizon**	211	58.7	218.9	262.3	135.2	364.3	108.9
	**C horizon**	215	61.2	251.2	262.1	191.3	363.5	90.1
**Cr**	**A horizon**	43.7	8.1	44.9	44	46.6	42.4	47.3
	**B horizon**	39.7	8.2	32.4	32.5	34.6	36.9	35.9
	**C horizon**	35.2	7.8	36.8	33.5	35.7	35.8	35.4
**Ag**	**A horizon**	0.454	0.072	0.523	0.25	0.194	0.291	0.394
	**B horizon**	0.432	0.083	0.362	0.261	0.147	0.488	0.138
	**C horizon**	0.362	0.062	0.522	0.29	0.197	0.412	0.089
**B**	**A horizon**	200	22	200	200	91.6	53.8	79.1
	**B horizon**	198	28	198	198	73	92.7	54
	**C horizon**	197	17	197	197	103	102.3	51.7
**Bi**	**A horizon**	13.1	0.53	8.47	2.66	1.68	1.33	2.66
	**B horizon**	7.9	0.48	6.21	6.62	3.51	3.36	1.39
	**C horizon**	7.6	0.23	6.79	6.65	3.67	2.97	1.14
**Co**	**A horizon**	11.7	5	20.2	10	10.5	7.7	13.2
	**B horizon**	6.8	4.8	11.3	12.3	14.5	5.9	14.5
	**C horizon**	5.8	5.1	11.2	11.4	13.8	6.7	12.2

Table S4 (*Continued*). Total concentrations of trace elements in three different soil layers

Element	Soil layer	TY-1	TY-2	TY-3	TY-4	TY-5	TY-6	TY-7
**Mo**	**A horizon**	1.29	1.13	1.21	0.89	1.07	0.87	1.11
	**B horizon**	3.45	0.83	2.19	4.08	2.02	3.26	3.28
	**C horizon**	2.36	0.98	2.68	4.48	2.23	3.87	3.37
**Sb**	**A horizon**	1.41	0.601	1.46	1.69	1.08	0.915	1.31
	**B horizon**	1.74	0.56	2.15	3.18	2.46	2.49	0.82
	**C horizon**	1.36	0.52	2.53	3.22	3.02	2.54	0.8
**Ti**	**B horizon**	4790.1	1708.2	4979	4850.2	4419	3776.8	4402.6
	**C horizon**	4789.2	1709.3	5672.4	4398.7	5256	5897	5120
	**A horizon**	5670.8	1880.5	5240	5512.5	5345.2	5643	2672
**V**	**B horizon**	60.5	16.9	64	60.2	69.8	53.2	72.5
	**C horizon**	81.6	16.5	77.8	118.3	86.2	134.6	123.7
	**A horizon**	79.8	16.7	94.6	119.6	104.7	129	43.8
**W**	**B horizon**	13	1.79	53.8	7.69	10.7	6.3	20.4
	**C horizon**	27.8	1.76	13.8	13.4	7.8	5.9	4.8
	**A horizon**	25.6	1.65	14.5	12.9	8.2	6.2	3.9
**Sn**	**B horizon**	35.9	5.8	34.6	28.9	16.9	23.1	19.2
	**C horizon**	35.2	3	38.4	28.8	18.7	17.9	14.2
	**A horizon**	38.6	2.8	34.8	34.6	19.2	18.2	5.6
